# Supplementary material for: Integration of cervical cancer screening into healthcare facilities in low- and middle-income countries: A scoping review
Source: PLOS Glob Public Health. 2024 May 14;4(5):e0003183. doi: 10.1371/journal.pgph.0003183 (PMC11093339; doi:10.1371/journal.pgph.0003183)
Supplement: S1 Table — (DOCX) [file pgph.0003183.s002.docx]

Supplementary Material 1**:** Search Terms Based on the Population, Concept, and Context (PCC) framework for MEDLINE.

| **P**-Population related terms | hospital* OR health cent* OR health post* OR healthcare instituti* OR health-care instituti* OR health instituti* OR clinic* OR Noncommunicable disease* care OR Non-communicable disease* care OR HIV care OR Female ward*  OR (Maternal and child health OR HIV OR sexual and reproductive health OR gyn?ecologic OR concept* OR Chronic care OR primary health care) adj5 (service*) OR (Family planning OR Tuberculosis OR TB* OR Primary care) adj5 (clinic*) OR (health OR healthcare OR health care) adj5 (facilit*) |
| --- | --- |
| **C**-Concept related terms | integrat* OR incorporation OR coordinat* OR co-ordinat* OR link* OR availab* OR readiness OR (cervi* cancer OR cervi* tumo?r OR cervic* neoplasm* OR cervical malignanc* OR tumo?r of the cervi* OR precancerous cell* of the cervi* OR cervi* Uteri*) adj5 (screen* OR test* OR examination* OR prevention OR control) |
| **C**-Context related terms (1) | Afghanistan* OR Algeria* OR Angola* OR Bangladesh* OR Benin* OR Bhutan* OR Bolivia* OR Burkina Faso* OR Burundi* OR Cabo Verde* OR Cambodia* OR Cameroon* OR Central African Republic OR Chad* OR Comoros* OR Congo OR Cote D'ivoire* OR Djibouti* OR Egypt* OR Arab Republic OR El Salvador* OR Eritrea* OR Eswatini* OR Ethiopia* OR Gambia* OR Ghana* OR Guinea* OR Guinea-Bissau* OR Haiti* OR Honduras* OR India* OR Indonesia* OR Iran OR Kenya* OR Kiribati* OR Korea OR Kyrgyz Republic OR Lao PDR OR Lebanon* OR Lesotho* OR Liberia* OR Madagascar* OR Malawi* OR Mali* OR Mauritania* OR Micronesia OR Mongolia* OR Morocco* OR Mozambique* OR Myanmar* OR Nepal* OR Nicaragua* OR Niger* OR Nigeria* OR Pakistan* OR Papua New Guinea* OR Philippines* OR Rwanda* OR Samoa* OR Sao Tome and Principe OR Senegal* OR Sierra Leone* OR Solomon Island* OR Somalia* OR South Sudan* OR Sri Lanka* OR Sudan* OR Syrian Arab Republic OR Tajikistan* OR Tanzania* OR Timor-Leste* OR Togo* OR Tunisia* OR Uganda* OR Ukraine* OR Uzbekistan* OR Vanuatu* OR Vietnam* OR West Bank and Gaza OR Yemen OR Zambia* OR Zimbabwe* OR Africa OR low resource OR Low-income countr* OR Lower-middle-income countr* OR Low-income nation* OR Lower-middle-income nation* OR low- and middle-income countr* |

1. Nada Hamadeh CVR, Eric Metreau & Shwetha Grace Eapen. WORLD BANK BLOGS [Internet]2022. Available from: <https://blogs.worldbank.org/opendata/new-world-bank-country-classifications-income-level-2022-2023>.
