# Supplementary material for: Integration of cervical cancer screening into healthcare facilities in low- and middle-income countries: A scoping review
Source: PLOS Glob Public Health. 2024 May 14;4(5):e0003183. doi: 10.1371/journal.pgph.0003183 (PMC11093339; doi:10.1371/journal.pgph.0003183)
Supplement: S2 Table — (DOCX) [file pgph.0003183.s003.docx]

Supplemental Material 2: JBI appraisal of analytical cross-sectional studies.

| Authors | Item 1 | Item 2 | Item 3 | Item 4 | Item 5 | Item 6 | Item 7 | Item 8 |
| --- | --- | --- | --- | --- | --- | --- | --- | --- |
| Anderson J *et al. 2015* | Yes | Yes | Yes | Yes | No | No | Yes | Yes |
| Bekolo CE *et al.2016* | Yes | Yes | Yes | Yes | Yes | Yes | Yes | Yes |
| Boddu A *et al.2021* | Yes | Yes | Yes | Yes | Not applicable | Not applicable | Yes | Yes |
| Cecilia Milford *et al*. 2017 | Yes | Yes | Unclear | Yes | Not applicable | Not applicable | Yes | Yes |
| Chawla PC *et al*.2014 | Yes | Yes | Yes | Yes | Unclear | Unclear | Yes | Yes |
| Claes P *et al*. 2003 | Yes | Yes | Yes | Yes | No | No | Yes | Yes |
| Colin Pfaf *et al. 2018* | Yes | Yes | Unclear | Yes | Not applicable | Not applicable | Yes | Yes |
| Elisabeth L. Vodicka *et al.2016* | Yes | Yes | Yes | Yes | Not applicable | Not applicable | Yes | Yes |
| Farida Selmouni *et al. 2023* | Yes | Yes | Yes | Yes | Yes | Not applicable | Not applicable | Yes |
| Jenell S. *et al.*2016 | Unclear | Yes | Unclear | Yes | Not applicable | Not applicable | Yes | Yes |
| Katie A. Ports *et al.* 2016 | Yes | Yes | Yes | Yes | Yes | Not applicable | Not applicable | Yes |
| Megan J *et al.* 2011 | Yes | Yes | Yes | Yes | No | No | Yes | Yes |
| Ninsiima et al. 2023 | Yes | Yes | Yes | Yes | Unclear | Unclear | Yes | Yes |
| Odafe, S. *et al.* 2013 | Yes | Yes | Yes | Yes | Unclear | Unclear | Yes | Yes |
| Padmaja R *et al.* 2013 | Yes | Yes | Yes | Yes | Yes | No | Yes | Yes |
| Phiri S *et al.* 2016 | Unclear | Yes | No | Yes | Not applicable | Not applicable | Yes | Yes |
| Prisca C *et al.*  2021 | Yes | Yes | Yes | Yes | Not applicable | Not applicable | Yes | Yes |
| Rupani M.P et al | Yes | Yes | Unclear | Yes | Yes | Yes | Unclear | Yes |
| Shiferaw *et al. 2015* | No | Yes | Yes | Yes | Not applicable | Not applicable | Yes | Yes |
| Wendimagegn NF. 2017 | Yes | Yes | Yes | Yes | Not applicable | Not applicable | Yes | Yes |
| Were E *et al.* 2010 | Unclear | Yes | Yes | Yes | Yes | Unclear | Yes | Yes |

Key

Item 1: Were the criteria for inclusion in the sample clearly defined?

Item 2: Were the study subjects and setting described in detail?

Item 3: Was the exposure measured in a valid and reliable way?

Item 4: Were objective, standard criteria used for measurement of the condition?

Item 5: Were confounding factors identified?

Item 6: Were strategies to deal with confounding factors stated?

Item 7: Were the outcomes measured in a valid and reliable way?

Item 8: Was appropriate statistical analysis used

Table 2: JBI appraisal for qualitative research.

| Authors | Item 1 | Item 2 | Item 3 | Item 4 | Item 5 | Item 6 | Item 7 | Item 8 | Item 9 | Item 10 | Score |
| --- | --- | --- | --- | --- | --- | --- | --- | --- | --- | --- | --- |
| Smit *et al.* 2012 | Yes | Yes | Yes | Yes | Yes | Unclear | Unclear | Yes | No | Yes | 7/10 |
| Elizabeth Roger *et al.* 2014 | Yes | Yes | Yes | Yes | Yes | Unclear | Unclear | Yes | Yes | Yes | 8/10 |
| EL Sibanda *et al*. 2015 | Yes | Yes | Yes | Yes | Yes | Unclear | Unclear | No | No | Yes | 6/10 |
| Kumakech *et al.* 2014 | Yes | Yes | Yes | Yes | Yes | No | Unclear | Yes | Yes | Yes | 8/10 |
| Kumakech *et al.* 2015 | Yes | Yes | Yes | Yes | Yes | No | No | Yes | Yes | Yes | 8/10 |
| Mpata PC *et al*. 2021 | No | Yes | Yes | Yes | No | No | Unclear | Yes | Yes | Yes | 7/10 |
| Akpan, E *et al*. 2023 | Yes | Yes | Yes | Yes | Unclear | No | No | No | Yes | Yes | 6/10 |

Key

Item 1: Is there congruity between the stated philosophical perspective and the research methodology?

Item 2: Is there congruity between the research methodology and the research question or objectives?

Item 3: Is there congruity between the research methodology and the research methods used to collect data?

Item 4: Is there congruity between the research methodology and the representation and analysis of data?

Item 5: Is there congruity between the research methodology and the interpretation of results?

Item 6: Is there a statement locating the researcher culturally or theoretically?

Item 7: Is the influence of the researcher on the research, and vice-versa addressed?

Item 8: Are participants, and their voices, adequately represented?

Item 9: Is the research ethical according to current criteria, or for recent studies, is there evidence of ethical approval by an appropriate body?

Item 10: Do the conclusions in the research report flow from the analysis, or interpretation of the data?
