# Supplementary material for: Integration of cervical cancer screening into healthcare facilities in low- and middle-income countries: A scoping review
Source: PLOS Glob Public Health. 2024 May 14;4(5):e0003183. doi: 10.1371/journal.pgph.0003183 (PMC11093339; doi:10.1371/journal.pgph.0003183)
Supplement: Attachment — Submitted filename: Reveiw report PLOSGPH-23-02587.pdf [file pgph.0003183.s004.pdf]

Summary

The study addresses a clearly important public health problem and explorers how the World Health Organization recommendations on integration of cervical cancer screening in health facilities are being implemented as well as facilitators and barriers. The goal of screening for cervical cancer is to find precancerous cervical cell changes when treatment can prevent cervical cancer from developing. The article is technically sound, has methodological rigor and is presented in an intelligible fashion.

Strengths

1. Use of multiple databases: Five electronic databases with peer-reviewed literature were used.
2. Scooping review conducted quality assessment of the journal articles.

Weaknesses

1. While the author identifies the burden by region, the author fails to link the high burden of Cervical cancer in SSA to the persistent infection with the human papillomavirus (HPV), regional differences in the cervical cancer burden are related to inequalities in access to vaccination, screening and treatment services, risk factors including HIV prevalence, and social and economic determinants such as sex, gender biases and poverty and the fact that women living with HIV are 6 times more likely to develop cervical cancer compared to women without HIV.
2. While the scooping review conducted quality assessment of the journal articles, those very poorly on quality were also included (inherent weakness of scooping reviews)
3. In the discussion section, the authors could increase relevance of the discussion by including contemporary guidance such as and the recent WHO recommendations on HPV DNA testing. Linking study finding and the literature with the contemporary recommendations for screening could increase the utility of the research findings and implications for scale up
4. In line 464, there is a mention for further research needed to understand the factors contributing to the acceptability of integrated services and develop strategies to enhance acceptance. Most (all) of the studies in the scooping review considered VIA as the main screening method for cervical cancer. With the recommendation on integration of HPV testing as part of the screening algorithms, the study does not provide insights on how this could impact the conclusions. This could be another area of further research

Recommendation:

I recommend approval with minor changes.

Evidence and Examples

Major issues:

1. Beyond the targets for Cervical cancer control by 2030 and the 90-70-90 targets; it is advisable that the authors also provide recent literature of the screening recommendations by WHO on Cervical cancer screening using DNA PCR. In one or another, inclusion of the recent WHO recommendations of HPV DNA PCR testing could have an implication on the policy considerations in the conclusion of this study (lines 491-503)
2. While quality of the articles was assessed, the analysis includes results from some of the poor-quality articles. However, this inherent methodological limitation/observation is not included in the limitation of the study.
3. In the discussions, provide insight into regional variations of cervical cancer burden and screening practices and the findings in the scooping review.

Minor issues:

4. Line 99 “published from were retrieved the year 2000’ has a typographical error caused by sequencing of words. Please correct to “ published were retrieved from the year….
5. Lines 218 and 227 refer to most women. Kindly quantify the word most by providing the ratio or percentage in the brackets to validate the choice of the word most
6. Please clarify: Line 301/302 does not show integration, and yet presented as a barrier to uptake.
7. Please clarify: Line 330/331 refers to fragmented services while referring to integration. Kindly clarify

Other Points

-None
