# Supplementary material for: Integration of cervical cancer screening into healthcare facilities in low- and middle-income countries: A scoping review
Source: PLOS Glob Public Health. 2024 May 14;4(5):e0003183. doi: 10.1371/journal.pgph.0003183 (PMC11093339; doi:10.1371/journal.pgph.0003183)
Supplement: Attachment — Submitted filename: Rebuttal letter_21.docx [file pgph.0003183.s005.docx]

**Rebuttal letter**

Dear Editor and reviewers,

We would like to express our deepest gratitude to you for taking the time to review our manuscript and providing us with very constructive comments. We truly appreciate it!

Journal Requirements:

1. We noticed you have some minor occurrences of overlapping text with the following previous publication(s), which needs to be addressed:

- <https://doi.org/10.1002/ijgo.12194>
-<https://doi.org/10.1186/s12913-023-09326-6>

In your revision ensure you cite all your sources (including your own works), and quote or rephrase any duplicated text outside the methods section. Further consideration is dependent on these concerns being addressed.

**Response:** Thank you for the valuable comment. We paraphrased some statements ((lines 51 & 52), (lines 64-66), and (lines 71-75))) in the introduction we felt overlapped with the mentioned references. We also added the reference number “12” in line 76 to strengthen the evidence.

2. Please amend your detailed Financial Disclosure statement. This is published with the article. It must therefore be completed in full sentences and contain the exact wording you wish to be published.

a. State the initials, alongside each funding source, of each author to receive each grant.
b. State what role the funders took in the study. If the funders had no role in your study, please state: “The funders had no role in study design, data collection and analysis, decision to publish, or preparation of the manuscript.”
c. If any authors received a salary from any of your funders, please state which authors and which funders.

If you did not receive any funding for this study, please simply state: “The authors received no specific funding for this work.

**Response:** Co-authors’ initials were not included because the grant was processed only through the principal investigator. This study is part of my (principal investigator) PhD work and the co-authors are my advisors. Accordingly, we tried to amend the financial disclosure statement as follows by including the initials of the PI.

This research was funded by the African Union Commission (AU), Addis Ababa, Ethiopia through the Pan African University Life and Earth Science Institute (PAULESI), University of Ibadan, Nigeria. The fund was processed through the principal investigator (RNK). The funders had no role in study design, data collection and analysis, or preparation of the manuscript. The views presented in the article are not necessarily expressing the views of the funding organization.

2. Please provide separate figure files in .tif or .eps format only and remove any figures embedded in your manuscript file. Please also ensure all files are under our size limit of 10MB.

For more information about figure files please see our guidelines:

<https://journals.plos.org/globalpublichealth/s/figures> 
<https://journals.plos.org/globalpublichealth/s/figures#loc-file-requirement>

**Response:** The figure’s format has been changed into. tif format based on the journal’s requirement, and attached as figure.

3. We have noticed that you have uploaded Supporting Information files, but you have not included a list of legends. Please add a full list of legends for your Supporting Information files after the references list.

**Response:** A list of legends has been added after the references

Finally, we added references for each article in the article summary table (Table 2)

**Review Comments to the Author**

Reviewer #1: This is nicely written and thorough review of the integration of cervical cancer screening into existing healthcare facilities in LMICs. As you will see in my review there are 2 major concerns: lack of clarity into the type of healthcare facility you are interested in (or is it all off them) and the manuscript is too long and repetitive.

You are interchanging and mixing “primary care facilities” and “healthcare facilities” which is causing confusion about what facilities you mean and are interested in. I think what you mean is: investigating CC screening into existing healthcare facilities that offer primary care services rather than offering CC screening as stand-alone services. This needs to be clarified early on (Introduction, line 63 would be a good place). Then be sure to choose and use the same language throughout (such as “healthcare facilities”). The abstract seems to imply you were comparing primary care facilities vs. all others. But Methods imply the type of facility does not matter.

**Response:** thank you for the constructive comment. You read what is in my mind! You are right the review aims to assess if there is cervical cancer screening integration into the pre-existing primary care services at any level of health facilities in low and middle-income countries. Not offering CC screening as a stand-alone service. Therefore, the aim of the review was to investigate if CCS was integrated into primary care services given at any level of health facilities and that’s why the objective of the review stated: “to map evidence related to integrating cervical cancer screening into existing primary care services/ not primary care facilities*/ and identify associated barriers and facilitators in LMICs”. The result in the abstract also described which pre-existed primary care services (HIV clinic, maternal and child health clinics, Family planning clinic, and so on) integrated CCS. As you have already mentioned, lines 63 to 73 describe WHO’s strategy for integrating CCS service integration into primary care packages at health facilities and the benefit of integrating the service with other services for women clearly stating it will help increase the screening uptake. But the meaning of integration might differ from article to article some of the articles show that CCS service was directly integrated with primary care services and giving the service together and the rest define integration if women come to the health facilities for any primary care service she will be counseled and referred to other primary clinics within the give health facility which integrated the CCS service. That’s why the method used at all levels of health facilities is to be included in the review.

**Abstract:**
The last sentence that “addressing the gaps could promote…integration into primary care services”. Are you considering the various settings listed in the sentence prior and the results to be “primary care services”? If yes, please clarify earlier. If not, then rephrase this sentence because the earlier presented information indicates it is successful in many settings.

**Response**: this is a very good view. We corrected the meaning of the conclusion per the given comment in a way that since CCS integration into different types of primary care services was found to have been available initially, addressing the identified barriers would help strengthen the successful integration of the service……...treatment outcomes.

**Introduction:**

Last paragraph: you state there are several studies on CC screening uptake but there is still room for more comprehensive evidence. Please elaborate on WHY there is still a need for comprehensive evidence. As stated, there is no gap for you to address…

**Response:** great! It is a good question, as you can see from the given references (14-19), all these articles could tell us about CCS uptake among women in various settings, where most of them investigated that there was low CCS service uptake. However, the studies fail to describe the integration of CCS services into the existing primary care services at health care facilities as WHO recommends (especially for LMICs) the service should be integrated to address women who are coming to the health set-up for any other primary care services or to make the service more accessible for the women. Therefore, this review intended to map evidence on the integration of the service into the health care facilities’ primary care services because one of the reasons for the low uptake of the service by women might be the unavailability of the service in an accessible way.

**Methods:**
Line 53-54: Is this reference Figure 1? If so, mention that.
**Response:** sorry, I do not understand this because line number 53-54 were not stated in the methods section. The stated line numbers were used in the introduction section.
**Results:**
Overall the Results section while nicely written is too long and repetitive. It reads like a Discussion section. To cut: (1) you should only present facts directly from the studies reviewed and remove opinions/conclusions/narrative from yourself—this is done in the Discussion. For example: Line 227: after “highlighting” is your conclusion; (2) Cut language. Simply state the fact as it relates to the Subheading without additional descriptive language (Example: Line 329-330 you do not need. Jump right into the barriers. A suggestion to consider is using bullet points in each subheading to briefly state each relevant finding; and (3) consider combining subheads where information is repetitive or could go together.

I suggest cutting the Sections on Lines 212-225. The first is not needed and the second is already covered by your table which shows the types of facilities and you describe the facilities you found in the first 2 paragraphs. Everything here is repetitive.

**Response:** we accept the comment that the statements in the result seem repetitive and we tried to amend some statements. However, the sections you mentioned in lines 212-225 are the main descriptions of the objective of the review. Because, since the objective also aimed to know which site or clinic in the health facilities integrated the CCS service. Therefore, with great apology, we kept them unchanged for now but if you are not comfortable with the inclusion of these sections, we are still ready to amend them. This is just to justify why we included them.

Line 252-253: Please add the reference for this study.

**Response:** The reference number has been added/corrected. We separately stated reference number 25 and 42.

Many of the results in each section are related to HIV clinics or HIV care, etc. There were many other clinics with CCS integration. Please make sure to mention those results. When you discuss HIV only clinics make sure the text is all together in that section. For example, in Section: Benefits of Integration of CCS Service into Healthcare Facilities...Please consider re-arranging this section to flow better. You present several points specific to HIV clinics and then mix in statements related to other types of clinical outside of HIV. I suggest trying a paragraph on the benefits to integrating into HIV clinics and then another on the non-HIV-specific information.

**Response:** Yes, you are right the benefits seem only related to HIV clinics. However, we have tried to review all the articles about the theme “benefit of CCS service integration” but most of the benefit of integration was mentioned in the articles where CCS service was integrated into HIV-clinics. As this review is a scoping review, we identified this as a gap for further research.

Numbers and statistics should be provided to support statements throughout. For example, Line 238 you say “most women included accepted…”. Please quantify “most” (i.e. 87% of women)

**Response:** the first sentence was written as the main statement of the paragraph but now we omitted it to avoid confusion.
Discussion
This section is nicely written and what I expect from a Discussion. But with the current Results section it simply repeats. Making changes to the Results will help.

**Response:** thank you so much for the constructive comment. Per the suggestion give in the result section, we tried to avoid redundancy of ideas.

Reviewer#2: Summary

The study addresses a clearly important public health problem and explorers how the World Health Organization recommendations on integration of cervical cancer screening in health facilities are being implemented as well as facilitators and barriers. The goal of screening for cervical cancer is to find precancerous cervical cell changes when treatment can prevent cervical cancer from developing. The article is technically sound, has methodological rigor and is presented in an intelligible fashion.
Strengths
1. Use of multiple databases: Five electronic databases with peer-reviewed literature were used.
2. Scooping review conducted quality assessment of the journal articles.
3. the data supports most of the conclusions and is available in the manuscript
Weaknesses

1. While the author identifies the burden by region, the author fails to link the high burden of Cervical cancer in SSA to the persistent infection with the human papillomavirus (HPV), regional differences in the cervical cancer burden are related to inequalities in access to vaccination, screening and treatment services, risk factors including HIV prevalence, and social and economic determinants such as sex, gender biases and poverty and the fact that women living with HIV are 6 times more likely to develop cervical cancer compared to women without HIV.
2. While the scooping review conducted quality assessment of the journal articles, those very poorly on quality were also included (inherent weakness of scooping reviews)
3. In the discussion section, the authors could increase relevance of the discussion by including contemporary guidance such as the recent WHO recommendations on HPV DNA testing. Linking study finding and the literature with the contemporary recommendations for screening could increase the utility of the research findings and implications for scale-up

**Response:** we found it a good comment. However, because of the difficulties involved with HPV and cytological screening methods, most low- and middle-income countries rely on visual inspection of the cervix with acetic acid (VIA) for screening. The global call to action CC elimination strategy guideline for LMICs developed by the WHO focuses on VIA for this region especially when it comes to primary care centers(1)( World Health Organization. A Global Strategy towards the Elimination of Cervical Cancer as a Public Health Problem, ISBN: 9789240014107)

4. In line 464, there is a mention for further research needed to understand the factors contributing to the acceptability of integrated services and develop strategies to enhance acceptance. Most (all) of the studies in the scooping review considered VIA as the main screening method for cervical cancer. With the recommendation on the integration of HPV testing as part of the screening algorithms, the study does not provide insights on how this could impact the conclusions. This could be another area of further research

**Response:** The majority of the articles included in the study showed that most of the CC screening was through VIA, and as the reviewer has already stated, the concept might be seen in future research. Besides, the aim of this review was to investigate if there is comprehensive evidence on CCS service integration into primary care services and barriers/facilitators at health care facilities in LMICs regardless of the screening method but as mentioned above most of the articles investigated that the screening method was VIA in most cases.
Recommendation:
I recommend approval with minor changes.

Evidence and Examples
Major issues:
1. Beyond the targets for Cervical cancer control by 2030 and the 90-70-90 targets; it is advisable that the authors also provide recent literature of the screening recommendations by WHO on Cervical cancer screening using DNA PCR. In one or another, inclusion of the recent WHO recommendations of HPV DNA PCR testing could have an implication on the policy considerations in the conclusion of this study (lines 491-503)

**Response:** Thank you for the dynamic view that enabled us to design another research question about methods of screening are available at health facilities in LMICs but describing screening methods was not the aim of the present review and we were not able to include the suggestion in the conclusion. The current review aimed to investigate comprehensive evidence on integration of the cervical cancer screening services into pre-existing services regardless of the method of screening or test. However, if you can further make the suggestion clearer, we are happy to include the mentioned literature in the introduction so that we will consider it in the conclusion too.
2. While quality of the articles was assessed, the analysis includes results from some of the poor-quality articles. However, this inherent methodological limitation/observation is not included in the limitation of the study.

**Response:** thank you so much. We included it in the limitation section.
3. In the discussions, provide insight into regional variations of cervical cancer burden and screening practices and the findings in the scooping review.

**Response:** we acknowledge the comment but we discussed it among authors and planned it to cover in another review since this will make the current review broader since we need to add another review objective that says regional variations of cervical cancer burden and screening practices.

Minor issues:
4. Line 99 “published from were retrieved the year 2000’ has a typographical error caused by the sequencing of words. Please correct to “published were retrieved from the year….

**Response:** corrected per the comment.
5. Lines 218 and 227 refer to most women. Kindly quantify the word most by providing the ratio or percentage in the brackets to validate the choice of the word most

**Response:** the first sentence was written as the main statement of the paragraph but now we omitted it to avoid confusion.

6. Please clarify: Line 301/302 does not show integration, and yet presented as a barrier to uptake.

**Response:** Yes, it seems the uptake. But, effectiveness of integrated cervical cancer screening includes different stakeholders including women. Even though there is an integrated screening service in the health facilities but if the women are not interested to take the service, we can’t talk about integrated CCS for the future so that it has its own role to be a barrier for integration policy.
7. Please clarify: Line 330/331 refers to fragmented services while referring to integration. Kindly clarify

**Response:** The point is that the integration definition differs in some cases because some articles consider integration if the service is given with the available primary service for example with family planning, outpatient department, and so on whereas some of them consider the integration of the screening service if the mother got screened when she comes to get another service but the CCS service might be in other clinics, within the health facilities, by the recommendation of the health professional who treated her in the previous clinic. Fragmentation of services might be mentioned because of the CCS services that would be performed after counseling or referral from the first clinic.

**Thank you once again for your valuable comments!**

1.	World Health Organization. A Global Strategy towards the Elimination of Cervical Cancer as a Public Health Problem, ISBN: 9789240014107. 2020.
